# Comparison of the efficacy of trastuzumab emtansine between patients with metastatic human epidermal growth factor receptor 2-positive breast cancers previously treated with combination trastuzumab and pertuzumab and with trastuzumab only in Japanese population

**DOI:** 10.1007/s12282-019-00949-4

**Published:** 2019-02-08

**Authors:** Shoko Noda-Narita, Akihiko Shimomura, Asuka Kawachi, Hitomi Sumiyoshi-Okuma, Kazuki Sudo, Tatsunori Shimoi, Emi Noguchi, Kan Yonemori, Chikako Shimizu, Yasuhiro Fujiwara, Kenji Tamura

**Affiliations:** 1 Department of Breast and Medical Oncology, National Cancer Center Hospital, 5-1-1, Tsukiji, Chuo-ku, Tokyo, 104-0045 Japan; 2 Department of Breast Medical Oncology, National Center for Global Health and Medicine, 1-21-1 Toyama, Shinjuku-ku, Tokyo, 162-8655 Japan

**Keywords:** Trastuzumab emtansine, Metastatic breast cancer, Human epidermal growth factor 2, Trastuzumab, Pertuzumab

## Abstract

**Background:**

Trastuzumab emtansine (T-DM1) has been approved since 2013 for patients with human epidermal growth factor receptor 2 (HER2)-positive metastatic breast cancer (MBC) who had received trastuzumab (Tmab) and taxane. However, no clinical trial has evaluated the efficacy of T-DM1 in those who have previously received pertuzumab (Pmab). This study aimed to compare the efficacy of T-DM1 between patients who had received Tmab and Pmab and those who had received Tmab only in Japanese population.

**Methods:**

We identified all patients with HER2-positive MBC who received T-DM1 between April 1, 2014 and February 28, 2017 in our institution. The patients were divided into the Tmab group (i.e., those who received only Tmab before T-DM1 treatment) and the Tmab/Pmab group (i.e., those who received Tmab and Pmab before T-DM1 treatment), and progression-free survival (PFS) and best response were compared between the two groups.

**Results:**

A total of 42 patients were enrolled for outcome analysis. The median follow-up period was 4.8 months, and the median number of prior chemotherapy regimens for metastatic disease before T-DM1 was 1 (range 1–2) in the Tmab/Pmab group and 2 (range 0–6) in the Tmab group. The median PFS was 2.8 months in the Tmab/Pmab group (95% confidence interval [CI] 1.7–4.8 months) and 7.8 months in the Tmab group (95% CI 5.5–15.9 months) (*p* = 0.0030). The best response was lower in the Tmab/Pmab group (11.1% vs. 25.0%).

**Conclusions:**

Patients with HER2-positive MBC who received Tmab and Pmab treatment before T-DM1 have fewer benefits from T-DM1.

## Introduction

The introduction of trastuzumab (Tmab), a monoclonal antibody targeting human epidermal growth factor receptor 2 (HER2), has substantially improved the prognosis of HER2-positive breast cancer. However, some patients with HER2-positive metastatic breast cancer (MBC) still experience progressive disease, indicating that such malignancy may be resistant to Tmab. To overcome this resistance, other HER2 targeting therapies, such as lapatinib (Lapa), pertuzumab (Pmab), and trastuzumab emtansine (T-DM1), were developed in the recent decade.

T-DM1 is an antibody-drug conjugate consisting of Tmab and the cytotoxic agent emtansine. It showed substantial benefits in progression-free survival (PFS) and overall survival (OS) compared with capecitabine and Lapa in patients with HER2-positive MBC who had previously received Tmab and taxane in the phase III EMILIA trial (median PFS, 9.6 months vs. 6.4 months; hazard ratio [HR] 0.65) [1, 2]. It was approved as second-line therapy for patients with HER2-positive MBC who previously received Tmab and a taxane by the United States Food and Drug Administration in February 2013 and in September 2013 in Japan. The phase 3 TH3RESA trial [3, 4] showed an improved efficacy of T-DM1 compared with that of treatment of physician’s choice for HER2-positive MBC patients who had received 2 or more HER2-directed regimens in the advanced setting (median PFS, 6.2 months vs. 3.3 months; HR 0.528). Moreover, an increasing number of patients are receiving T-DM1 as second- or later-line chemotherapy in the metastatic setting. Concurrently, the approval of Pmab as first-line treatment in combination with taxane and Tmab has changed the standard treatment for HER-2 positive MBC [5, 6]. This resulted in a situation in which patients to be treated with T-DM1 in clinical practice are different from those enrolled in the pivotal trial (EMILIA trial). Although the EMILIA trial and other clinical trials of T-DM1 did not enroll patients who previously received Pmab, at present, many patients with HER-2 positive MBC have received Pmab before T-DM1 treatment.

Dzimitrowicz et al. [7] evaluated the efficacy of T-DM1 in those who previously received T-DM1 in clinical practice and compared it with that in clinical trials. Their study demonstrated a lower tumor response rate (TRR) of 17.9% and shorter median duration of therapy of 4.0 months (95% confidence interval [CI] 2.7–5.1 months) in 78 patients, of which 96.7% had previously received both Tmab and Pmab in the metastatic setting. This study made us hypothesize that the limited efficacy of T-DM1 is related to the Pmab exposure. However, the resistant mechanisms of T-DM1 and other HER2-targeted drugs are not fully investigated [8], and there are no mechanisms that can totally explain this disappointing result of T-DM1.

The resistant mechanism of T-DM1 is related to DM1 metabolism, including poor internalization of HER2-T-DM1 complex, intracellular and endosomal trafficking of the complex, and multi-drug resistance transporters accelerating efflux pumps [9]. Other resistant mechanisms to T-DM1 include activation of the PI3K pathways by neuregulin b1, which can trigger the formation of HER2-HER3 heterodimers [10, 11]. The resistance mechanisms of Pmab/Tmab therapy are poorly investigated, but some have been identified, including rapid dimerization of HER3/EGFR, resulting in the activation of posterior proliferative pathways [12].

There are no similarities in the resistant mechanisms of Pmab and T-DM1. Moreover, it is difficult to explain the reason why T-DM1 would have disappointing result in patients treated after Tmab/Pmab combination therapy in preclinical studies. The difference in resistant mechanisms of Tmab/Pmab combination chemotherapy and chemotherapy only containing Tmab need to be determined.

Given that only few studies have estimated the efficacy of T-DM1 in patients with Pmab exposure [12, 13], the current study aimed to assess the efficacy of T-DM1 for patients with HER-2 positive MBC according to their prior treatment history. Toward this goal, we conducted a retrospective study that compared the PFS and best response between patients who previously received Tmab and Pmab and those who received only Tmab in Japanese population.

## Patients and methods

### Patients

We used electronic pharmacy records to identify the patients who received T-DM1 after the diagnosis of HER2-positive MBC in the National Cancer Center Hospital (NCCH) between April 1, 2014 and February 28, 2017. The patients who had previously received neither Tmab nor Pmab and patients who had received T-DM1 as prior treatment were excluded. The enrolled patients were divided into the Tmab group (i.e., those who received only Tmab before T-DM1 treatment) and the Tmab/Pmab group (i.e., those who received Tmab and Pmab before T-DM1 treatment). The patients’ electronic medical records were reviewed manually and the following data were collected: patient characteristics including date of birth, sex, performance status (PS) at the time of starting T-DM1 therapy, and tumor characteristics, including estrogen receptor (ER), progesterone receptor (PgR), and HER2 status of the primary tumor and metastatic tumor, if reassessed, and the site of metastases at the time of starting T-DM1 therapy.

ER, PgR and HER2 receptor status was determined by a local pathologist according to the 2010 and 2013 American Society of Clinical Oncology/College of American Pathologists guidelines, respectively. Treatment history was also collected, including prior neo-adjuvant and adjuvant chemotherapy and endocrine regimens, and prior chemotherapy and endocrine regimens in the metastatic setting. The dates of first courses of Tmab, Pmab and T-DM1 were also recorded. Tumor response was determined by the treating physician, although the clinical assessment was reviewed to evaluate the tumor response. The date of death was also recorded as applicable.

The present study was approved by the NCCH Institutional Review Board. Written informed consent was waived owing to the retrospective nature of the study.

### Efficacy

The efficacy of T-DM1 in patients who previously received Tmab and Pmab in the metastatic setting was compared according to PFS and best response with that in patients who previously received only Tmab. Progressive disease (PD), in this study, was defined as the time when the tumor was assessed radiologically PD according to RECIST version 1.1, and PFS was calculated as the length of time after the start of the T-DM1 treatment until PD.

### Statistical analysis

Kaplan–Meier method was used to estimate the median PFS and 95% CIs, and between-group comparisons were performed via log-rank tests. We used a Cox proportional-hazards model to estimate the HR. Best response was assessed according to RECIST version 1.1. Tumor response rate (TRR) and disease control rate (DCR), which are defined as the percentage of patients who achieved complete response (CR), partial response (PR) and stable disease (SD) continuing over 6 months, were compared between the two groups using Chi-square test, and a two-sided *p* value < 0.05 was considered significant.

## Results

### Patients characteristics

**Fig. 1:**
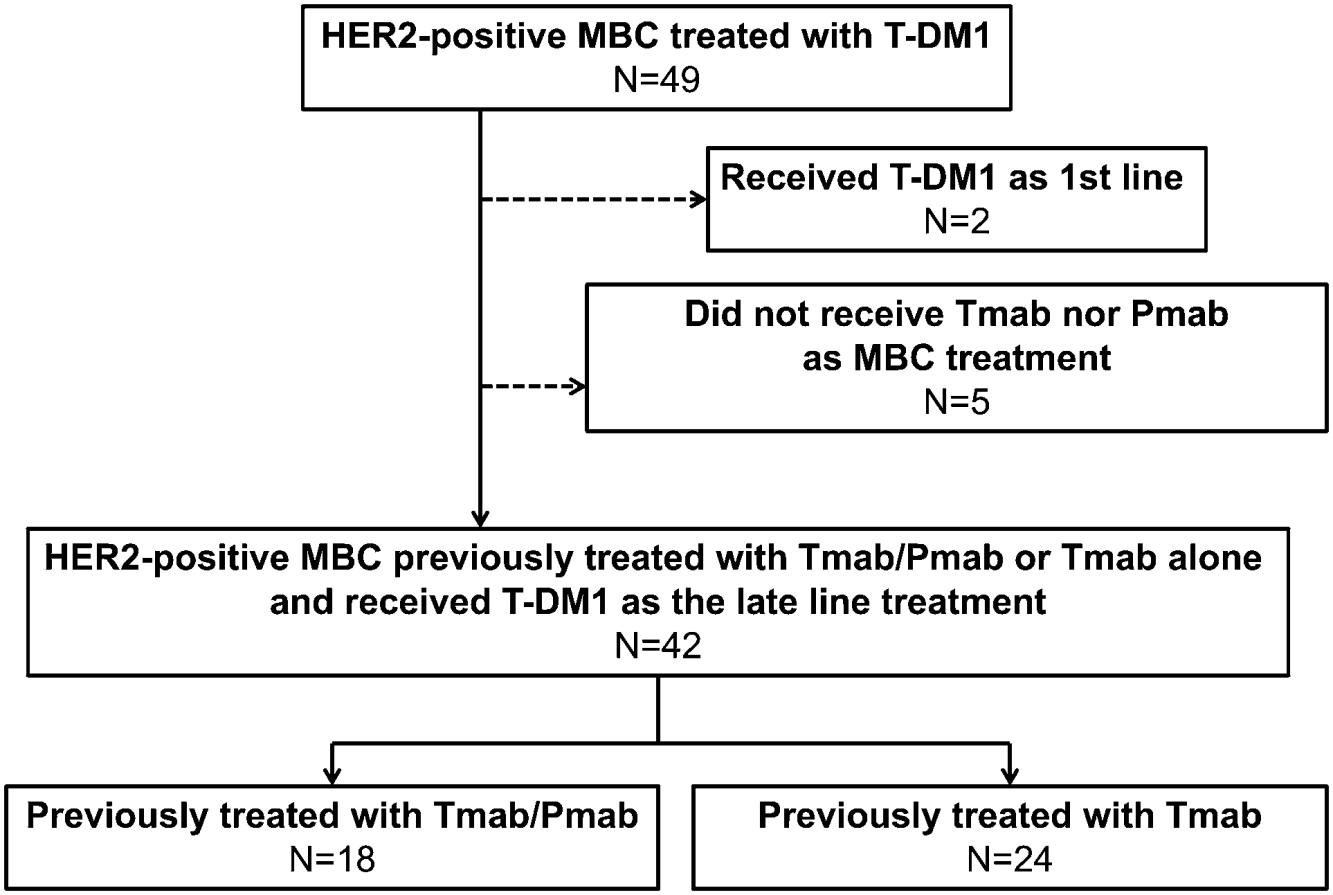
Patient recruitment flow chart. *HER2* human epidermal growth factor receptor 2, *MBC* metastatic breast cancer, *T-DM1* trastuzumab emtansine, *Tmab* trastuzumab, *Pmab* pertuzumab

A total of 49 patients who received T-DM1 in NCCH between April 1, 2014 and February 28, 2017 for HER2-positive MBC were identified. Two patients who had received T-DM1 as prior treatment and five patients who had previously received neither Tmab nor Pmab were excluded. Finally, 42 patients were assessed in this study (Fig. 1). The median age was 57 years (range 30–74 years), and 41 of the 42 patients (98%) had PS 0–1. A total of 33 of the 42 patients (79%) had visceral disease, which was defined as brain, pulmonary, pleural, or liver metastasis, at the start of the T-DM1 treatment. The Tmab/Pmab group comprised 18 patients, while the Tmab group comprised 24 patients. There was no difference in the baseline characteristics between the two groups. The median number of chemotherapy regimens previously received in the metastatic setting was 1 (range 1–2) in the Tmab/Pmab group and 2 (range 0–6) in the Tmab group. Eleven of the 18 patients (61%) and 10 of the 24 patients (42%) in the Tmab/Pmab and Tmab groups, respectively, had hormone receptor-positive MBC (Table 1). Sixteen of the 18 patients (89%) in the Tmab/Pmab group received Pmab as combination chemotherapy with docetaxel (DTX) and Tmab, and patients in the Tmab group received Tmab as combination chemotherapy with paclitaxel, DTX, capecitabine, or vinorelbine. A total of 23 patients in the Tmab group received T-DM1 after disease progression in metastatic setting, while the other one patient received T-DM1 after the relapse to adjuvant chemotherapy including Tmab.

**Table 1:** Patient characteristics

Characteristics	Tmab/Pmab group (*n* = 18)	Tmab group (*n* = 24)	*p* value
Age	53 (43–73)	60 (30–74)	0.2501
ECOG PS			0.3971
0	13 (72)	13 (54)	
1	5 (28)	10 (42)	
2	0 (0)	1 (4)	
Metastatic site			0.8394
Visceral	13 (72)	18 (75)	
Non-visceral	5 (28)	6 (25)	
Hormone receptor status			0.4740
ER + and/or PgR+	11 (61)	10 (42)	
ER− and PgR−	7 (39)	14 (58)	
Number of prior chemotherapy regimens	1 (1–2)	2 (0–6)	0.0020
Previous exposure to HER2-targeted therapy			
Tmab	18 (100)	24 (100)	
Duration (months)	15.1 (3.5–32.5)	30.4 (1.8–90.2)	
Lapa	0 (0)	6 (25)	
Duration (months)		6.79 (1.8–10.8)	
Pmab	18 (100)	0 (0)	
Duration (months)	7.7 (2.1–32.5)		
Total duration of HER2-targeted therapy	15.1 (3.5–32.5)	31.0 (1.8–90.2)	

Data are presented as median (range) or number (%). Visceral disease: with brain, pulmonary, pleural, or liver metastasis

*Tmab* trastuzumab, *Pmab* pertuzumab, *ECOG PS* Eastern Cooperative Oncology Group performance status, *ER* estrogen receptor, *PgR* progesterone receptor, *Lapa* lapatinib

### Efficacy

**Fig. 2:**
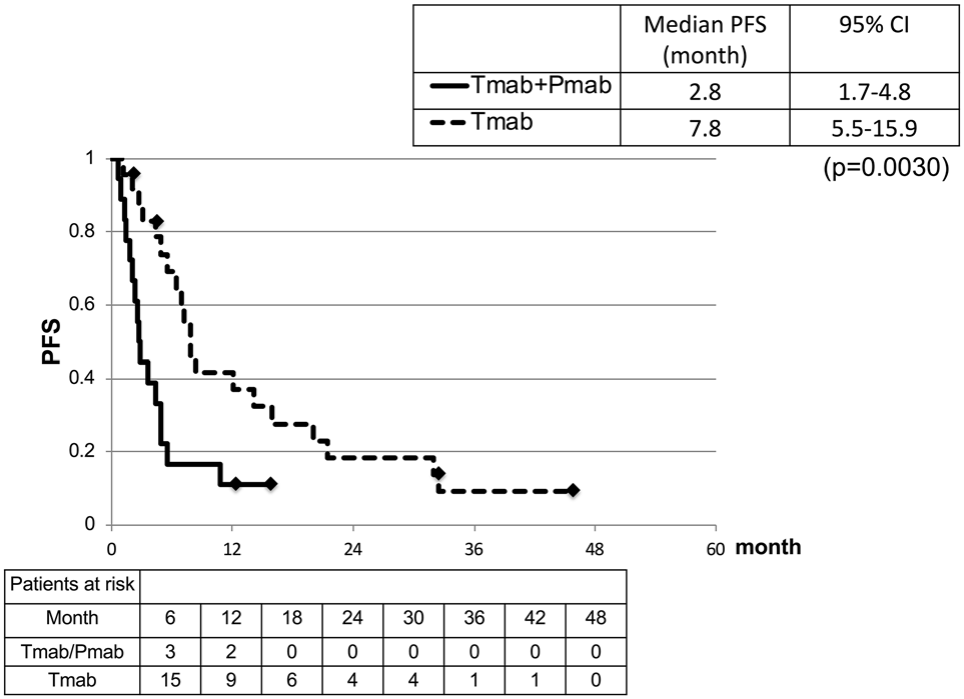
Progression-free survival

The median follow-up time of T-DM1 treatment was 2.8 months (range 0.7–12.2 months) in the Tmab/Pmab group and 7.5 months (range 1.1–45.7 months) in the Tmab group. PFS was significantly shorter in the Tmab/Pmab group than that in the Tmab group (2.8 months [95% CI 1.7–4.8 months] vs. 7.8 months [95% CI 5.5–15.9 months]; *p* = 0.0030) (Fig. 2). The HR was 2.83 (95% CI 1.37–5.89), and 3.07 (95% CI 1.46–6.54) adjusted by PS, metastatic site (visceral or non-visceral) and hormone receptor status using a multivariable regression model.

The TRR was 11.1% (2/18 patients) in the Tmab/Pmab group and 25.0% (6/24 patients) in the Tmab group (*p* = 0.2566) (Table 2). The DCR including PR and SD over 6 months was 16.7% (3/18 patients) in the Tmab/Pmab group and 62.5% (15/24 patients) in the Tmab group. T-DM1 treatment was discontinued in 40 patients due to PD (*n* = 38, 95%), diagnosis of another tumor (transformed follicular lymphoma; *n* = 1, 2.5%), and because of treatment-related anorexia (*n* = 1, 2.5%).

**Table 2:** Best response

	Tmab/Pmab group (*n* = 18)	Tmab group (*n* = 24)	*p* value
Tumor response rate, *n* (%)	2 (11.1)	6 (25.0)	0.2566
Disease control rate, *n* (%)	3 (16.7)	15 (62.5)	0.0030
Partial response, *n* (%)	2 (11.1)	6 (25.0)	
Stable disease, *n* (%)	6 (33.3)	15 (62.5)	
Progressive disease, *n* (%)	10 (55.6)	3 (12.5)	

Disease control rate: including partial response and stable disease for more than 6 months

*Tmab* trastuzumab, *Pmab* pertuzumab

## Discussion

This is the first report to demonstrate the efficacy of T-DM1 in patients who had previously received Tmab and Pmab compared with patients who had received only Tmab in Japanese population. Our study showed the shorter median PFS of 2.8 months and lower TRR of 11.1% in the Tmab/Pmab group compared to the Tmab group. Although the difference is not statistically significant, the result of T-DM1 in Tmab/Pmab group was disappointing, considering the result in pivotal studies.

Compared with the results of the EMILIA trial, the median PFS of 7.8 months in the Tmab group in the current study is slightly shorter than that in the clinical trial (9.6 months) [1], but it is acceptable considering that the patients in our study received T-DM1 as later-line regimen than in the clinical trial. However, the median PFS of 2.8 months in the Tmab/Pmab group is substantially shorter than that in the EMILIA trial (9.6 months) [1], and even shorter than that in the TH3RESA trial (6.2 months) [3]. Although our study is a retrospective evaluation of patient outcomes in routine clinical practice, the patient characteristics suggested that the patients in the Tmab/Pmab group in our study had better PS. Moreover, they received T-DM1 in former chemotherapy line compared with that in the TH3RESA trial. Further, the outcome after Pmab exposure was obviously worse than that of patients who had only received Tmab in the current study.

The first retrospective study which investigated the efficacy of T-DM1 after Pmab combination therapy was conducted by Dzimitrowicz et al. [7], and they showed shorter median duration of therapy (4.0 months [95% CI 2.7–5.1 months]) in patients who had received Pmab. This study was a retrospective single-arm study, and the authors considered duration of therapy as surrogate indicator of PFS. They reported that the shorter duration of therapy might be caused by the retrospective nature of the research and the relatively high percentage of de novo stage IV patients (44%). And also, they demonstrated 30.8% of prolonged disease control rate over 6 months, and mentioned the importance of overall benefit of T-DM1 in patients who received prior Pmab. Our study included only 24% of de novo stage IV patients, and the lower efficacy of T-DM1 in the Tmab/Pmab group in our study might be a result of the concordance of resistant mechanisms in T-DM1 and Tmab/Pmab combination therapy. Of the importance, only 16.7% of patients in our study remained response more than 6 months, which indicate that fewer population who received prior Pmab would receive the overall benefit from T-DM1 treatment than previously reported.

There were two other retrospective studies conducted in Italy, which compared PFS of T-DM1 between patients who had received Pmab and those without Pmab exposure [13, 14]. They also showed the shorter duration of PFS in patients who had received Pmab in second line treatment (3 months [95% CI 2–4 months] vs. 8 months [95% CI 4–12 months] [13] and 5.0 months [95% CI 4.3–5.7 months] vs. 11.0 months [95% CI 7.8–14.2 months] [14]). The study reported by Fabi et al. [14] provided propensity score-matched sample with perfect match for age and PFS at first-line, and it also showed a shorter PFS in patients treated with Pmab (5.0 months [95% CI 4.3–5.7 months] vs. 11.0 months [95% CI 7.3–14.0 months]). However, all institution involved in the previous three retrospective studies were in Western countries, and our study was the first to compare the outcomes between patients who had previously received Pmab and patients without Pmab exposure in Asian population. Our study showed relatively shorter PFS in both Tmab and Tmab/Pmab group than the previous two studies from Italy probably because the study included patients in latter lines.

Of importance, our study was a retrospective examination in routine clinical practice, and it might limit the interpretation of the results. In clinical trials, eligibility is usually defined according to patient characteristics, and the patients are more homogeneous than those in retrospective studies. In this study, the patients in the Tmab group and the Tmab/Pmab group had different chemotherapy history, and this might have resulted in different sensitivity to HER-2 targeted therapies. A total of 16 of the 18 patients (88.8%) in the Tmab/Pmab group had previously received DTX as a combination cytotoxic agent, while only 41.7% of the patients in the Tmab group had received DTX. The patients in the Tmab/Pmab group had only previously received a median of 15.1 months of HER-2 targeted therapy, while the patients in the Tmab group had a much longer median duration of exposure to HER-2 targeted therapy at 31.0 months. Particularly, the patients included in our study were those who received T-DM1 between April 1, 2014 and February 28, 2017, while Pmab combination therapy of Tmab/Pmab/DTX was first used in Japan in September 2013. Thus, the patients treated using this regimen had shorter follow-up period than those who were treated using the previous first-line regimen that did not include Pmab. As such, this study might have tended to enroll patients who had lower sensitivity to HER2-targeted therapy in the Tmab/Pmab group, yielding shorter median PFS than that in the control group in the TH3RESA trial (3.3 months) [3].

Although the resistant mechanisms for HER2-targeted therapy are yet to be thoroughly investigated, the difference in resistant mechanisms for each of the HER-2-targeted drugs might have caused the substantial variation in the efficacy of T-DM1, as shown in this study. Resistance to T-DM1, which occurs after Tmab/Pmab combination therapy, might be associated with the dimerization of HER2 and other EGFRs, and this might interfere with the binding of the T-DM1 ligand to tumor cells.

To determine the cause of the result of minimal efficacy of T-DM1 in our study, further investigations of the resistant mechanisms of T-DM1 and other HER-2 targeted therapy are needed. Moreover, to confirm the efficacy of T-DM1 after Pmab exposure, an ongoing prospective study (Clinicaltrials.gov identifier: NCT01835236) may estimate the efficacy of T-DM1 in such patients. This randomized phase 2 trial assigns previously untreated patients with MBC to either Tmab/Pmab with chemotherapy group or Tmab/Pmab without chemotherapy group, and both treatment regimens are followed by T-DM1. This will be the first prospective clinical trial to evaluate the efficacy of T-DM1 for patients after Pmab therapy, and the efficacy of T-DM1 in this trial could be compared to the efficacy that the EMILIA and TH3RESA trials have shown. Although it is difficult to accurately conclude at this point, patients with HER2-positive MBC who received Tmab and Pmab treatment before T-DM1 may receive fewer benefits from T-DM1.

## Data Availability

The datasets generated during and/or analyzed during the current study are not publicly available due to individual privacy, but are available from the corresponding author on reasonable request.

## Abbreviations

CI: Confidence interval
CR: Complete response
DCR: Disease control rate
DTX: Docetaxel
EGFR: Epidermal growth factor receptor
ER: Estrogen receptor
HER2: Human epidermal growth factor receptor 2
HR: Hazard ratio
Lapa: Lapatinib
MBC: Metastatic breast cancer
NCCH: National Cancer Center Hospital
OS: Overall survival
PD: Progressive disease
PFS: Progression-free survival
PgR: Progesterone receptor
Pmab: Pertuzumab
PR: Partial response
PS: Performance status
SD: Stable disease
T-DM1: Trastuzumab emtansine
Tmab: Trastuzumab
TRR: Tumor response rate
